# A case report of metastasis of malignant mesothelioma to the oral gingiva

**DOI:** 10.1186/1758-3284-3-21

**Published:** 2011-04-22

**Authors:** Stephanie Moser, Marc Beer, Georg Damerau, Heinz-Theo Lübbers, Klaus W Grätz, Astrid L Kruse

**Affiliations:** 1 University Hospital Zurich, Department of Craniomaxillofacial and Oral Surgery, Plattenstrasse 15, CH-8032 Zurich, Switzerland

**Keywords:** Mesothelioma, metastasis, soft tissue

## Abstract

**Introduction:**

Metastatic mesothelioma to the oral cavity arises from the pleura or peritoneum and distant hematogenous metastases are seen in more than half of cases but only a few cases are reported to the oral cavity.

**Case:**

A 75 year old male suffering from metastatic mesothelioma presents an hyperplasia of the attached gingiva. Malignant mesothelioma is a rare tumour arising from pleura, pericardium or peritoneum.

**Conclusion:**

This article highlights the importance of biopsy and histopathological diagnosis of oral lesions especially in case of a malignant history.

## Introduction

Malignant pleural mesothelioma is a malignant neoplasm of mesodermal origin and arises from multipotential mesothelial or subserosal cells of the pleura, pericardium and peritoneum. There are different forms: epitheloid (60%), sarcomatoid (10-20%) and biphasic patterns (20-30%). Metastases to the oral cavity are very rare [1,2]. They are more common in the jaw bones than the soft tissue. The most common sites for men are the lungs ( 27%), kidneys (13%) and skin (13%) - for women, the breast (24%) and genital organs (17%), followed by bone (10%) and kidney (10%) [1]. The reason for this gender-dependent metastatic pattern has not completely been elucidated yet. In 90% of all pleural mesothelioma, asbestos-association due to occupational exposure is reported. The tumour is found in patients in the fifth and sixth decades. The latency between exposure and manifestation takes approximately 20-40 years. Occurrence of the malignant disease typically carries an average survival rate of 9-12 months [3].

A patient with a history of malignant pleural mesothelioma with metastatic disease to the attached gingiva is presented.

### Patient

A 75-year-old man was referred from his private dentist to the Department of Oral Surgery, University of Zurich with a painless growth of the attached gingiva in the disto-buccal region of tooth 35. It had been present for 6 weeks and had increased in size during this period of time.

**Figure 1:**
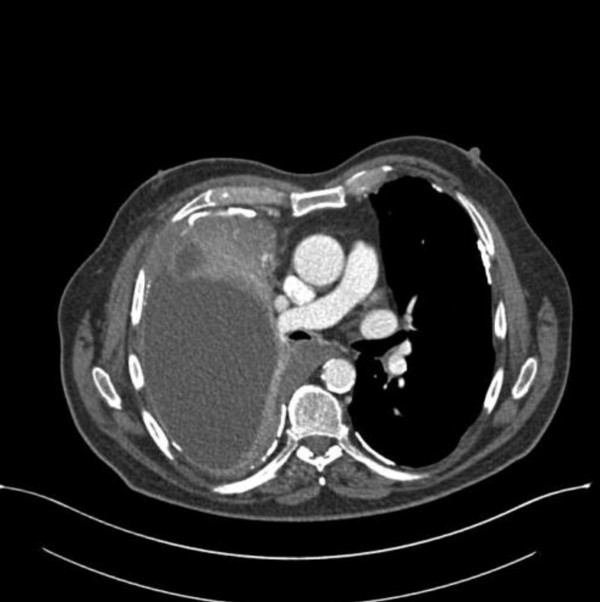
Chest CT presenting the mesothelioma

An epitheloid mesothelioma was diagnosed 2 years earlier (Figure 1) and treated with chemotherapy. The patient commenced the drug Alimta^® ^(Pemetrexed) with palliative intent. He had had a significant asbestos exposure during his working life as an electrician. His medical history was otherwise unremarkable. He was a non-smoker.

**Figure 2:**
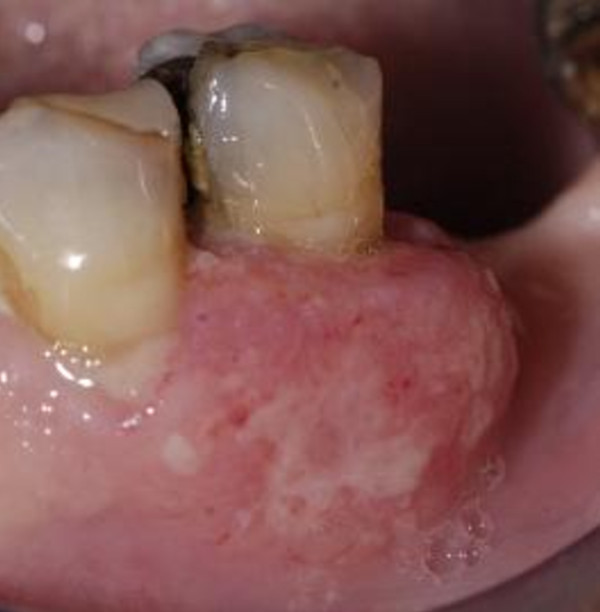
Hyperplasia of the attached gingiva showing the soft tissue metastasis

Extraorally, neither swelling nor lymphadenopathy was discernible. The innervation of the N.trigeminus was balanced on both sides. Oral examination showed an exophytic, compact and in parts ulcerous swelling surrounding the premolar tooth 35 (Figure 2). The lesion measured approximately 1.5 cm by 1.0 cm. The affected tooth was of grade one mobility, no pathological results in sensitivity and percussion were found and there was no objective paraesthesia in the distribution of the left mental nerve.

**Figure 3:**
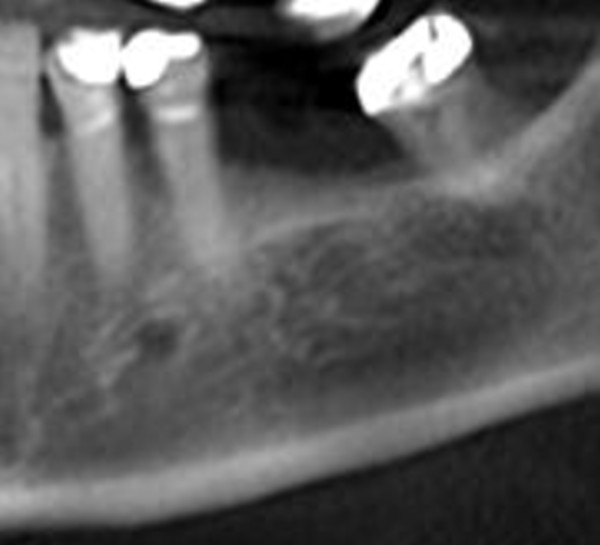
Orthopantomogram is showing no bony destruction

**Figure 4:**
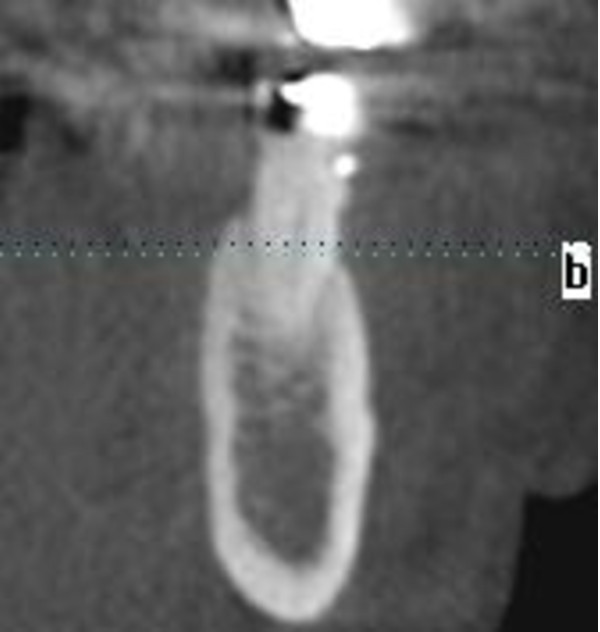
Digital volume tomography sagittal is showing no changings in bony architecture

Radiological examination (orthopantomogram and digital volume tomography) of the lower jaw revealed no destruction of the bony architecture (Figure 3, 4). There were no changes to the structure of the compacta or spongiosa.

An incisional spindle-shaped biopsy with a thread-mark was carried out under local anaesthesia. The sample measured 10 × 5 mm including both normal and modified gingiva. During the biopsy an unusual blood flow was conspicuous. The second premolar was not extracted.

Histology and immunohistochemistry confirmed the diagnosis of a metastasis of the pleural mesothelioma.

### Diagnostic considerations

Clinically, soft tissue metastases typically present as sessile or nodular masses that characteristically resemble hyperplastic reactive lesions, such as pyogenic granuloma or giant cell granuloma [4]. Differential diagnosis, such as other benign tumours of the oral mucosa including lipoma, myxoma, neurofibroma, schwannoma, leiomyoma and various forms of epulis, have to be considered. Murray et al. [5] reported a case where the clinical appearance was most suggestive of a fibroepithelial polyp localized on the dorsum of the tongue. Therefore, excisional biopsy of the lesion was arranged to confirm the diagnosis histologically.

### Histology

Grossly, the specimen was a polypous lesion measuring 0,5 × 0,4 cm, protruding from a mucous membrane spindle with a white cut face.

**Figure 5:**
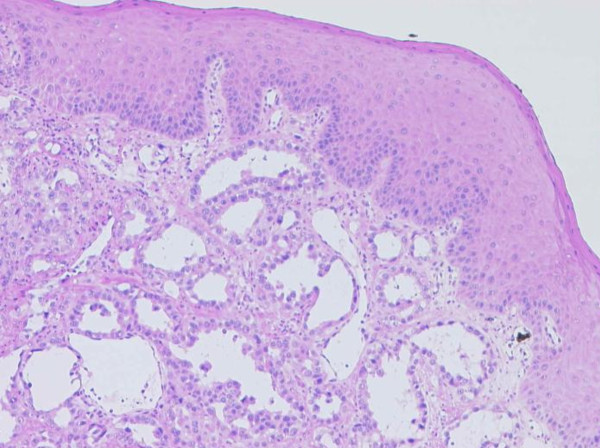
**Conventional H&E morphology (50× and 100×) showing squamous mucosa with tubular tumour infiltrates**.

**Figure 6:**
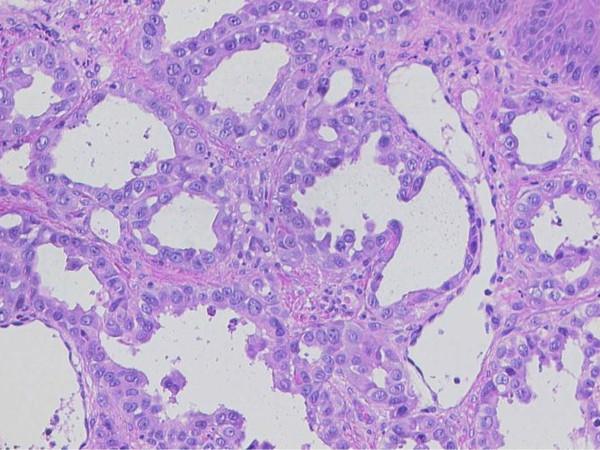
**Conventional H&E morphology (50× and 100×) showing squamous mucosa with tubular tumour infiltrates**.

On microscopy, a submucous infiltrate of a neoplasm with marked cell atypia and a glandular growth pattern was seen (Figure 5 &6).

**Figure 7:**
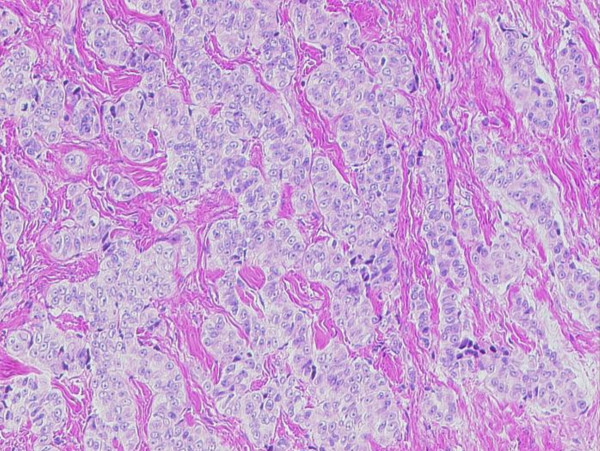
**Conventional H&E (100×) morphology of pleural Biopsy taken in 2008 showing an infiltrate with a trabecular architecture**.

In comparison, the pleural biopsy taken two years previously, when malignant mesothelioma was originally diagnosed in the patient, showed very similar cytology but a more trabecular architecture with only occasional tubular formations (Figure 7).

Conventional morphology ruled out clinical differential diagnoses such as pyogenic granuloma, epulis, drug-induced gingival hyperplasia or soft tissue tumours.

Likely differentials were metastasis of epitheloid mesothelioma or adenocarcinoma, primarily of the lung.

**Figure 8:**
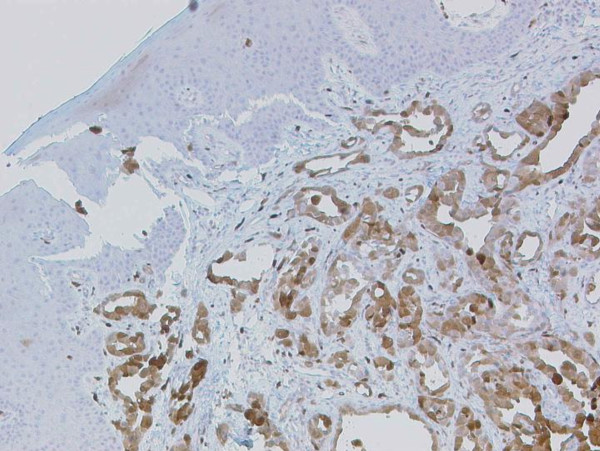
**Calretinine stain (50×), strong positivity of the tumour cells for this mesothelial marker**.

Calretinin, a mesothelial marker turned out to be strongly positive in tumour cells on immunohistochemical staining (Figure 8), substantiating diagnosis of an epitheloid mesothelioma metastasis. Positive staining for cytokeratins 5/6 and negative staining for Ber-EP4 further helped to distinguish it from adenocarcinoma.

## Discussion

Metastases of a malignant mesothelioma are very rare but have previously been described. An English literature review (Table 1) revealed six cases on the tongue, four (including the present report) the attached gingiva, one case the bone and one the floor of the mouth in only male patients with a previous diagnosis of mesothelioma. The median age was 61.25 years with a median time from diagnosis to development of the oral metastasis was 12.4 months.

**Table 1:** Reported cases of soft tissue metastases in mesothelioma (1993 - 2010)

Case	Age at time of occurence	Sex	Previous/existing diagnosis of mesothelioma	Site of primary mesothelioma	time from diagnosis to development of oral metastasis in months	Localisation	Publication
						**Tongue**	
1	73	m	Yes	Pleural	24	right lateral	1993 [13]
2	52	m	Yes	Pleural	24	right lateral	2003 [14]
3	71	m	Yes	Pleural	14	right lateral	2003 [15]
4	70	m	Yes	Pleural	9	left lateral	2005 [4]
5	68	m	Yes	Pleural	5	anterior central	2007 [16]
6	46		Yes	Pleural	8	dorsum	2010 [5]
						**attached gingiva**	
1	45	m	Yes	Pleural	12	lingual 36	1993 [13]
2	48	m	Yes	Pleural	4	regio 35-36	1993 [17]
3	63	m	Yes	Pleural	11	regio 35 buccal	2002 [8]
4	75	m	Yes	Pleural	24	regio 35 buccal	2010^current report^
						**Bone metastasis**	
1	53	m	Yes	Pleural	2	regio 38	2003 [7]
						**Floor of mouth**	
1	71	m	Yes	Pleural	12	right	2010 [18]

Metastatic tumours to the oral cavity and jaws are uncommon lesions and represent about 1% of all metastases in humans. Primary breast carcinomas are the most common types of tumour to metastasize to the jaw bones while primary lung carcinomas are the most common types of tumour to metastasize to the oral soft tissue [6]. These oral metastases represent about 1% of oral malignances and there are more cases reported arising in the jaw bones than in the oral soft tissues with a ratio of approximately 2:1 [7]. Most of the soft tissue metastases occur on the gingiva and alveolar mucosa, with an additional 27% involving the tongue. It seems that the presence of teeth has a crucial effect on the oral site preference of metastases. In dentulous patients, 79% exhibited their metastases in the attached gingiva, whereas in edentulous patients, metastastic lesions were equally distributed between the tongue and alveolar mucosa [8]. In this case, the primary tumour was histologically classified as an epitheloid type. Law et al. reported that the epithelial type has a tendency to invade directly and cause lymphatic metastases similar to carcinoma, whereas the sarcomatous type tends to metastasize hematogenously like sarcoma [7].

Only a small number of reports for metastases in the oral cavity of a malignant pleura mesothelioma were found in the literature (Table 1). 12 cases were found in all. The age-range of the male-only patients is 45 - 75. The tongue (6 cases) seems to be the preferred site for metastases, followed by the attached gingiva (4 cases) and only 1 case is published which affects either the lower jaw or the floor of the mouth. One possible explanation for the tendency towards the tongue and the attached gingiva might be the rich capillary network, especially where chronically inflamed gingiva trap malignant cells and the fragmented basement membranes of proliferation capillaries allow easier penetration by malignant cells than in more mature blood vessels [9]. The tongue also is well vascularized and it has been postulated that this may provide a good setting for malignant cells [10].

Several differential diagnosis should be taken into consideration. An enlargement or overgrowth of the gingiva is a recognized complication that has been associated with the administration of phenytoin, some calcium channel blockers, and cyclosporine [11]. However, in most of these cases, hyperplasia is general rather than localized, although there are still exceptions.

As with the present patient, pemetrexed with a platinum agent is the regime of first choice in chemotherapy for mesothelioma [12]. There are many known side effects, but no publication was found which describes an effect to the oral soft tissue.

This article highlights the importance of biopsy and histopathological diagnosis of oral lesions especially in case of a malignant history. Furthermore, a regular screening for oral neoplasms is indispensable. Prognosis for patients with malignant mesothelioma is very poor. The majority of patients are incurable and the treatment is aimed at palliation. Having consulted our patient's oncologist, one decided to limit the therapy to regular recalls and an alteration of the chemotherapy drugs but to avoid surgical treatment with resection of the tumour because of reduced life expectancy.

## Consent

Written informed consent was obtained from the patient for publication of this case report and accompanying images. A copy of the written consent is available for review by the Editor-in-Chief of this journal.

## Competing interests

The authors declare that they have no competing interests.

## Authors' contributions

SM carried out the retrospective study and drafted the manuscript, MB participated in the design of the study, AK participated in the design and coordination of the study. GD performed corrections of the draft. HTL and KWG performed corrections of the draft and assisted in designing the study. All authors read and approved the final manuscript.
